# Rapeseed oleogels based on monoacylglycerols and methylcellulose hybrid oleogelators: Physicochemical and rheological properties

**DOI:** 10.1016/j.fochx.2024.101520

**Published:** 2024-05-28

**Authors:** Mehdi Naderi, Aman Mohammad Ziaiifar, Ladan Rashidi, Seid Mahdi Jafari

**Affiliations:** a Department of Food Process Engineering, Gorgan University of Agricultural Sciences and Natural Resources, Gorgan, Iran; b Department of Food and Agricultural Products, Food Technology and Agricultural Products Research Center, Standard Research Institute (SRI), Karaj, Iran; c Halal Research Center of IRI, Iran Food and Drug Administration, Ministry of Health and Medical Education, Tehran, Iran

**Keywords:** Hybrid oleogels, Monoacylglycerols, Methyl cellulose, Emulsion-coating approach

## Abstract

In this study, we investigated the combined impact of monoacylglycerol (MAGs) and methylcellulose (MC) on the production of hybrid oleogels. Since cellulose derivatives are inherently hydrophilic substances, they require dissolution in oil through an emulsion-coating method. Therefore, we developed a hybrid oleogel utilizing MAGs and MC. Initially, a hybrid oleogelator was created by blending an aqueous MC solution into fully melted MAGs to form MC in water-in-MAGs emulsions with varying MC/MAG ratios, followed by drying. Subsequently, the resulting oleogelator was mixed with rapeseed oil to produce oleogels, and their properties were compared with oleogels produced solely with MAG oleogelator. The findings indicated that the obtained oleogelator did not significantly impact the oxidation of the oleogels. Additionally, there was no notable difference observed in the induction period of crystallization and the crystallization rate of the oleogels. Microscopic images revealed that the hybrid oleogel structured with a 30:70 ratio of MAGs and MC contained the lowest liquid phase percentage. In terms of rheological assessment, the hybrid oleogels exhibited solid-like behavior, consistent with polarized light microscopy (PLM) images. Furthermore, based on the three-interval thixotropic test (3-ITT), the hybrid oleogels displayed higher recovery compared to the control sample.

## Highlights

• Emulsion-coating approach was applied to prepare a hybrid oleogelator.
• Methylcellulose enhanced the impact of monoacylglycerols as crystalline particles.
• Hybrid oleogels showed a behavior similar to solids.
• Hybrid oleogels with stronger texture were obtained at higher temperature.
• Hybrid oleogels showed a higher recovery (%) compared to the MAG10.

## 1 Introduction

Oleogels are fat-based systems formed through dissolving oleogelators (OLGs) in a liquid oil. In recent years, oleogelation of edible oils has been introduced as an effective strategy to minimize the trans fatty acids (TFAs) and saturated fatty acids (SFAs) due to its structured 3D gel networks formed by OLGs which entrap the liquid oil (Naderi, Farmani, & Rashidi, 2016; Patel & Dewettinck, 2016; Singh, Auzanneau, & Rogers, 2017). Generally, they are heated above their melting point and then cooled to form a thermoreversible gel (Hwang & Winkler-Moser, 2020). Recent studies have explored various methods for producing oleogels. Wei et al. (2022) investigated the impact of oleogelation of monoacylglycerols (MAGs) on the textural, oil binding capacity (OBC), and rheological properties of walnut oil oleogel. Kim, Hwang, Jeong, and Lee (2021) studied the use of oleogels with binary mixtures for low-saturated filler creams as substitutes for fatty products. Additionally, Patel (2017) suggested the production of microcapsules coated with methyl cellulose on palm stearin to create hybrid oleogels.

In terms of the direct and/or indirect dispersibility in edible oils, compounds can be divided into two groups: hydrophilic and hydrophobic. No complicated approaches are needed to incorporate the hydrophobic compounds into the oil due to their organic nature and they are simply heated to be dissolved in the organic phase (Patel, 2017). They include fatty acids such as 12-hydroxystearic acid, phospholipids such as lecithin, fatty alcohols such as stearyl alcohol and partial glycerides (monoacylglycerols (MAGs) or diacylglycerols (DAGs)) (Naderi et al., 2016). Also, hydrophilic compounds such as hydroxypropyl methylcellulose (HPMC) and methylcellulose (MC) can be used as the structuring agents in the production of oleogel systems. These compounds are produced synthetically by replacing hydroxyl groups on the cellulose structure with methoxy and hydroxypropyl groups (Grover, 2020). MC is naturally surface-active due to the hydrophobic substituents present on its cellulose framework, (Patel & Dewettinck, 2015). In general, MC with higher methyl ether groups tends to form stronger gels and requires lower temperatures to dissolve (Patel et al., 2014a, Patel et al., 2014b). While MC is hydrophilic in nature, it exhibits a marked surface activity at oil/water interfaces due to the presence of methyl groups (Grover, 2020). However, this polymer is not directly dispersible in the organic solutions. The main problem with hydrophilic compounds is its insolubility in the oil phase. Hence, indirect method is to be used to solve them in the oil (Patel & Dewettinck, 2015; Yousefi, Rajaei, Nateghi, Rashidi Nodeh, & Rashidi, 2023). Ethyl cellulose is the only cellulose derivative that dissolves in oil only at high temperatures (>120 °C) (Haj Eisa, Laufer, Rosen-Kligvasser, & Davidovich-Pinhas, 2020), causing accelerated oxidative reaction (Patel & Dewettinck, 2016). Specific approaches must be therefore employed to dissolve these compounds under milder conditions. In general, these approaches are referred to as indirect methods. The most common indirect processes to make building blocks include emulsion-templated and foam-templated (Bayés-García et al., 2015; Espert, Salvador, & Sanz, 2020; Patel, 2017). These frameworks can trap oil in their porous structure. While in the emulsion-templated process, the moisture of prepared emulsion is directly removed by a freeze dryer or hot air to form a desired oleogel system (Espert et al., 2020), in the foam-templated process, a foam system is firstly produced by dissolution of the hydrophilic OLGs in an aqueous solution and it is then dried. Edible oil is then incorporated into this porous structure using a mixer (shearing process) and lastly a cryogel is produced (Patel & Dewettinck, 2015). The main difficulty with these approaches is that they require relatively complicated processes which restrict their applicability. Accordingly**,** several researchs were performed on the applicability of cellulose derivatives in oleogels systems (Ahmadi, Tabibiazar, Roufegarinejad, & Babazadeh, 2020; Qi, Li, Zhang, & Wu, 2021; Rodríguez-Hernández, Pérez-Martínez, Gallegos-Infante, Toro-Vazquez, & Ornelas-Paz, 2021).

The objective of this study was to produce hybrid OLGs based on MAGs and MC with different ratios using an emulsion-coating approach. Correspondingly, cellulose derivatives are coated on a hydrophobic compound, creating a stable emulsion. It is therefore feasible to produce the hybrid oleogels with extremely high applicability. In this context, the novelty of this research lies in the use of MAG as the main base in the production of a hybrid oleogelators.

## 2 Materials and methods

MC with a viscosity of 1500 mPa.s from the Sigma-Aldrich company (St. Louis, MO, USA), MAGs from Behbood Asia Powder Co. (Mashhad, Iran) and rapeseed oil from Pars Vegetable Oil Co. (Pishva, Varamin, Iran) were obtained.

### 2.1 Fabrication of hybrid OLGs

**Fig. 1:**
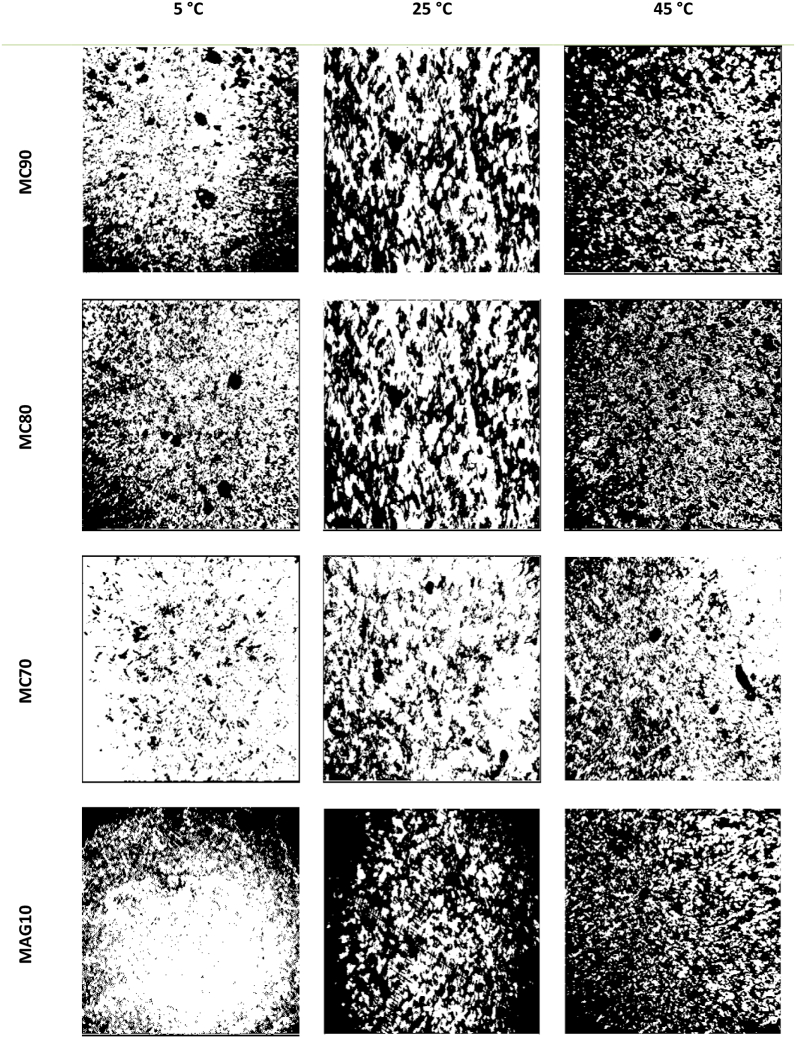
Polarized light microscopy (PLM) of hybrid oleogels at 5 °C, 25 °C and 45 °C at 10× magnification. MC90, MC80, and MC70 are oleogel samples made using oleogelators derived from a W/O emulsion with MAGs: MC ratios of 30:70, 20:80, and 10:90, respectively; MAG10: Control sample.

An emulsion-templated method was used to produce a stable emulsion based on MC and MAGs (Patel et al., 2014a, Patel et al., 2014b). Briefly, the MAGs were firstly heated to 55 °C up until they melt to become a liquid using a heating magnetic stirrer (IKA-Werke GmbH & Co. KG, Germany). A 2% *w*/w aqueous solution of MC was then prepared and added slowly to 1500 g of fully-melted MAGs to make a W/O emulsion with ratios of 30:70, 20:80 and 10:90 of MAGs: MC. W/O emulsion was then stirred using an ultra-high shear mechanical stirrer (WiseStir HS-30D, Daihan Scientific Co, Seoul, Korea) at 50 to 55 °C until the formation of a white, stable, and uniform emulsion (Fig. 1SA). The obtained emulsion was then placed in a freezer (−18 °C) for 1 h. To obtain a flowable hybrid OLGs powder (with a maximum 5% moisture content), the emulsion was finally dried at the temperature of 30 to 35 °C using an oven (VDL23, Binder GmbH, Germany) for 24 h until a thin layer of OLGs forms, indicating that their moisture content is negligible.

### 2.2 Production of the hybrid oleogels

The dried OLGs (in a proportion of 10% *w*/w) were dissolved in rapeseed oil at 62 °C (the melting temperature of hybrid OLGs) for 30 min using a heating magnetic stirrer (IKA-Werke GmbH & Co. KG, Germany) to form the MC70, MC80 and MC90 hybrid oleogels (Fig. 1SB). Control sample (MAG10) was prepared by dissolving of MAGs powder in rapeseed oil (10% (*w*/w) at the same temperature of 62 °C for 10 min using the heating magnetic stirrer.

### 2.3 Fatty acid composition of MAGs

Preparation of fatty acid methyl esters was done according to the AOCS Ce 2–66 method and its analysis was performed by the gas-liquid chromatography (GC) (Agilent (USA) model 6100) according to the AOCS 1Ce-91 method (Brühl, 1997). The GC was equipped with a split injection (1:100) and a flame ionization detector. The length, internal diameter and thickness of the capillary column CP -Sil 88 were 100 m, 0.25 mm and 0.25 μm, respectively. Nitrogen was selected as a carrier gas and the temperatures of detector and injector were adjusted at 300 and 280 °C, respectively. The oven temperature was adjusted as isothermal at 190 °C. Accordingly, the amounts of C12:0. C14:0, C16:0, C18:0, C18:1, TFAs and SFAs of MAGs were obtained 0.02 ± 0.1%, 2.5 ± 0.1%, 96.5 ± 0.1%, 0.3 ± 0.1%, 0.3 ± 0.1%, 0.0, 99.5 ± 0.1%, 0.2 ± 0.1%, respectively.

### 2.4 Solid fat content (SFC)

SFC was measured according to the sequential and direct method of AOCS 16b-93 at different temperatures of 5, 10, 20, 30, 40, and 50 °C with a magnetic resonance spectrometer (pNMR) (Bruker, Minispec mq20, Germany) (Brühl, 1997). The samples were completely melted at 70 °C. The tubes containing samples were then placed at 0 °C for one hour. The samples were placed into a water bath for 35 min at any desired temperature, the SFC (%) of the hybrid oleogels was recorded (three sets of experiments were conducted).

### 2.5 Crystallization kinetics

The crystallization kinetics were quantified using the described method by Naderi et al. (2016). Accordingly, the hybrid oleogels were melted at 80 °C. The samples were then placed in a water bath at the desired temperature and its SFC (%) was read using a pNMR instrument (Bruker, Minispec mq20, Germany) at regular intervals (every 5 s) until no change was observed. Since the crystallization curve of the hybrid oleogels has a sigmoidal shape, the modified Gompertz model (Eq. (1)) was applied to fit it. Using the coefficients, A, B and M obtained in this equation, the induction period of crystallization (Eq. (2)) and the crystallization rates (Eq. (3)) were calculated.

***(1)*** $$y=A+Ce^{-e^{-B\left(t-M\right)}}$$

***(2)*** $$\text{Induction period of crystallization}=M-\frac{1}{B}$$

***(3)*** $$\text{Crystallization rate}=\frac{B*C}{e}$$

where *A* is the asymptotic *Y* (crystallization content) when *t* (time) decreases indefinitely, *C* is the asymptotic *Y* that occurs when *t* increases indefinitely, and *B* is the relative rate of crystallization at *M*, where *M* is time, at which the absolute crystallization rate is maximum.

### 2.6 Oxidative stability test

According to the method No. AOCS 12B-92, the oxidative stability of the hybrid oleogels was determined at 110 °C using a Rancimat instrument (Herisau, Switzerland). The sample flow rate and air velocity were adjusted 2.5 ± 0.05 g/s and 2.5 mL/s, respectively (Brühl, 1997).

### 2.7 Rheometry

The rheological analyzes were carried out using the Anton Paar Rheometer (MCR 301, Anton-Paar, GmbH, Graz, Austria) where the geometry of parallel plates was 40 mm in diameter. The rheological tests including a temperature ramp test (with a heating ramp of 2 °C/s) and frequency sweep tests (at 0.1 to 100 Hz) were conducted and subsequently, dynamic moduli, including elastic modulus (G'), viscous modulus (G") and complex modulus were obtained. The oscillatory tests were carried out in the linear viscoelastic range. The temperature ramp test was conducted at 5 °C to 55 °C and the frequency sweep tests were carried out at a constant temperature of 5 °C (Naderi et al., 2016). A Peltier system was applied as heating system. In addition, a 3-interval thixotropy test (3-ITT) was carried out for the hybrid oleogels. To this end, they were subjected to different cycles of low and high shear rates (0.1 and 10 s^−1^ respectively) at a constant temperature of 5 °C and the data were illustrated as viscosity (Pa. s) vs time (Patel et al., 2014a, Patel et al., 2014b). Also, recovery (%) of the oleogels were calculated using Eq. 4. *V*_*i*_ and *V*_*30*_ represent viscosity immediately after the first stress and after the first 30 s of deformation, respectively.

***(4)*** $$\text{Recovery}\;\left(\%\right)=\frac{V30}{\mathrm{Vi}}*100$$

### 2.8 Study of the hybrid oleogels microstructure using polarized light microscopy

The Olympus BX50 light polarizing microscope (Olympus, Tokyo, Japan) was applied to study the hybrid oleogels microstructure. Specific weight of the hybrid oleogels were firstly fully melted and then stored at 5, 25 and 40 °C overnight. The microstructure of hybrid oleogels was then explored at the desired temperature with magnification of 10*×* (Naderi et al., 2016). Using PLM images, liquid phase fraction (%) (which is representative of liquid phase (%)) was calculated using ImageJ software version 1.46 (NIH, Bethesda, MD).

### 2.9 Statistical analysis

All data are reported as the mean ± standard deviation. The experiments were performed in a fully randomized design. A one-way ANOVA analysis was conducted using SPSS V.16 software to determine differences among the samples. Following this, Duncan's test was utilized to evaluate significance at a *p*-value of <0.05.

## 3 Results and discussion

### 3.1 Microstructure of hybrid oleogels

One of the common and fast techniques to study the fat microstructure is to investigate the microscopic images obtained by polarized light, in which, the amorphous segment of a substance is appeared as a dark sheet. Accordingly, for the oils without fat crystals, the recorded image was a completely black sheet due to the lack of birefringence properties. In fact, the poorly structured fat shows an image with darker areas. Using polarized light microscopy (PLM) images, the firmness of fats can be also observed as they always have a combination of liquid and solid phases. While the image captured from liquid phase is as a dark sheet due to lack of refractive index, the images of the solid phase appears as a bright or white sheet. Two parts (black and white) are therefore recorded in the polarized light images.

Based on this fact, the hybrid oleogels with white part as predominant have less liquid phase (%) and vice versa (Narine & Marangoni, 1999; Sánchez-Becerril et al., 2018). The PLM images of the hybrid oleogels at 5, 25 and 45 °C is shown in Fig. 1. Increasing the temperature from 5 to 45 °C slightly decreases the strength of the samples. The PLM of the hybrid oleogels demonstrated that the liquid phase (%) of the samples increased in the emulsions with lower proportion of MC solution (Fig. 1). In general, as mentioned in Table 1S, higher MAGs in the hybrid OLGs decreased the liquid phase (%) (*P* < 0.05). Furthermore, at higher temperatures, the hybrid oleogels such as MC70 had less liquid phase (%) than the MAG10 sample (*P* < 0.05). Patel and Dewettinck (2015) accordingly stated that the cellulose derivative-based oleogels exhibited a higher birefringence due to the semi-crystalline nature of polymer strands which form a bond between the fat crystals resulting in a higher firmness. While the MAG10 sample was experienced a strong structural modification at higher temperature of 45 °C, the hybrid oleogels retained their structure more optimally, possibly due to the reinforcement effect of the MC on the MAGs. MAGs (as a crystalline particles) create a thermally reversible gel, and MC (as a polymer strand) increased the strength of the hybrid oleogels by coating the surface of MAGs. In general, the role of crystalline fat particles such as MAGs is to create heat reversible and mouth feel properties. On the other hand, polymer strands increase the strength of the fats (Patel & Dewettinck, 2015). The higher firmness of oleogels can be attributed to the emulsion-coating method used in their fabrication. According to Liu and Binks (2022), the foamability and foam stability of crystalline particles, such as sorbitan esters, are influenced by several factors. Aeration in the one-phase molecular region at high temperatures allows for faster adsorption of surfactant monomers to the air-oil surface, facilitating efficient air incorporation. The high viscosity of oleogels, compared to oil solutions, limits the amount of air bubbles that can be incorporated into the continuous oil phase. Rapid cooling induces local crystallization of surfactant molecules at bubble surfaces and in the continuous phase, enhancing interfacial elasticity of air bubbles, preventing bubble dissolution and coalescence, and triggering gelation of the continuous oil to prevent drainage, coarsening, and coalescence. Our results are according to Li and Liu (2023). They reported the oil diffusion was slower in the oleogel fabricated with cocoa butter and MAGs due to the tighter connection between them. Also, Sun et al. (2022), expressed the change trend of OBC of MAG-based oleogel aligns with hardness levels, indicating a link between mechanical strength and OBC. Therefore, Oleogels with higher mechanical strength typically have a higher OBC.

### 3.2 Oxidative stability of hybrid oleogels

Under accelerated conditions, the secondary oxidation products such as aldehydes and ketones are broken down into third oxidation products (formic acid). This compound enhances the electrical conductivity, which signifies the completion of the oil oxidation process. This mechanism is the basis for the measurement of the induction period of oxidation (IP_OX_) by the Rancimat instrument. In general, saturated fats have a longer IP_OX_ compared to unsaturated fats (O'brien, 2008; Pan, Tang, Dong, Li and Zhang, 2021). As summarized in Table 1S, there was no significant difference between the IP_OX_ treatments (*P* > 0.05). Accordingly, higher MC and lower MAGs in the OLGs have no effect on the IP_OX_. In line with our findings, the MAG-based hybrid oleogels possessed higher IP_OX_. It was suggested by Paradiso et al. (2014) that partial triacylglycerols such as MAGs, in negligible concentrations, increase the oxygen solubility by lowering the surface tension. On the other hand, at higher concentrations (>5%), these compounds show the antioxidant activity. In fact, MAGs were layered on the surface of the oil, which resulted in a decrease the oxygen contact with the oil. These results were in agreement with the observations of Naderi, Farmani, and Rashidi (2018) and Gomes, Caponio, Bruno, Summo, and Paradiso (2010) who suggested that the greater MAGs levels led to the higher IP_OX_. In this context, Naderi et al. (2018) reported that the rise in OSI due to the addition of MAGs may be attributed to the composition of fatty acids, particularly the higher levels of saturated fatty acids. This can delay the formation of formic acid, ultimately resulting in an increase in OSI. The other reason behind of increase in OSI could be attributed to the applied method (high shear method). Hereof, nanoemulsions play a vital role in reducing absorption variability and providing protection against oxidation and hydrolysis. This is because oil-in-water nanoemulsions are resistant to air and water exposure (Garcia et al., 2022).

### 3.3 Solid fat content of hybrid oleogels

The SFC (%) significantly influences the hardness of fat in the temperatures ranged from the refrigerator to the mouth (O'brien, 2008). Accordingly, SFC shows the spreadability of a fat at refrigerator temperature (5 °C), liquefaction of a fat at room temperature (25 °C), and the its mouthfeel at 35 °C (Saberi, Kee, Oi-Ming, & Miskandar, 2011). The SFC (%) of hybrid oleogels is given at 5 to 45 °C in Table 2S. Although SFC (%) of hybrid oleogels slightly increased at higher proportion of MAGs in the OLGs composition, no significant difference was observed between the SFC (%) of hybrid oleogels at the same temperature (*P* > 0.05). On the other hand, as the temperature increased from 5 to 45 °C, the SFC (%) showed a marked decrease (*P* < 0.05). Since MAGs are highly saturated compounds, they can increase SFC (%). Furthermore, MAGs inherently have a very wide melting range due to the presence of free hydroxyl groups in their structure. Saberi et al. (2011) reported that the SFC curves of MAG/DAG-based oils have a flatter slope, indicating the strength of its structure in different temperature ranges and making it suitable for the production of margarine and shortening.

### 3.4 Crystallization kinetics of hybrid oleogels

**Fig. 2:**
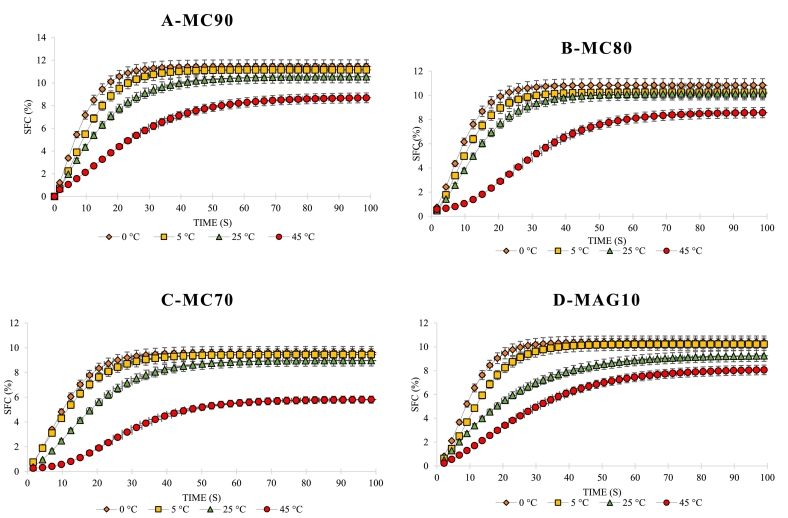
Crystallization curves of hybrid oleogels at 0, 5, 25 and 45C; MC90 (A), MC80 (B), MC (70), MAG10 (D). MC90, MC80, and MC70 are oleogel samples made using oleogelators derived from a W/O emulsion with MAGs: MC ratios of 30:70, 20:80, and 10:90, respectively; MAG10: Control sample. SFC: solid fat content.

Fat-containing foods are affected by the crystallization process. This process is directly associated to the firmness, the sensory evaluation and the physical properties. One of the crucial parameters in the formation of crystal nuclei is the crystallization induction period (IP_cryst_). This parameter is the time during which the primary nuclei of fat crystals form. IP_cryst_ is inversely relevant to the rate of nucleation and/or rate of crystallization (O'brien, 2008). As shown in the Fig. 2 the crystallization curve of the hybrid oleogels had a sigmoid (S) shape. Under isothermal conditions, this curve had 3 different phases. The first phase showed the IP_cryst_ of the hybrid oleogels before increasing in SFC (%), the second phase showed the crystallization rate of the hybrid oleogels due to the exponential increase of SFC (%) and the third phase or static phase showed the completion of the hybrid oleogel crystallization. Table 3S shows the IP_cryst_ and crystallization rate at 0, 5, 25 and 45 °C. Obviously, an increase and a decrease in the IP_cryst_ and crystallization rates were respectively shown at higher temperatures. A slight increase in the IP_cryst_ and decrease in the crystallization rate of the hybrid oleogels were observed at higher MC ratio in the OLGs (*P* < 0.05). In addition, a less difference between treatment and the control was observed at 0, 5 and 25 °C (P < 0.05). However, at higher temperature of 45 °C, the control showed a shorter IP_cryst_ than other treatments (about 10 s). MAG/MC-based hybrid oleogels resulted in instantaneous crystallization. Although the hybrid oleogels had a longer IP_cryst_ than the control sample, this parameter was about 20 s even at 45 °C. This finding agreed well with earlier studies performed by Patel (2017) who reported that the crystallization process of the hybrid oleogels was delayed. The presence of MC as a polysaccharide may impact the interactions within the complex system, potentially affecting the gelation behavior. (Wang, Virgilio, Wood-Adams, & Heuzey, 2017). In this context, the molecular interactions between these two ingredients (MC and MAGs) play a significant role in achieving a specific texture and functionality in resultant oleogels. Our data are contrary to those reported by Li and Liu (2023). The crystallization mechanism of cocoa butter-oleogel suggests that oleogels hinder cocoa butter crystallization, thereby delaying polymorphic transformation and improving the stability of chocolate bloom. In this context, Zhang, Xu, Tang, and Li (2023) revealed that the complex gel fabricated with glyceryl monostearate (GMS) had higher crystallinity, resulting in better thermal stability and mechanical properties compared to glyceryl monolaurate-based oleogels at the same MAG level. They reported Increasing MAG content resulted in denser three-dimensional gel network structures in oleogels, leading to improved mechanical properties, thermal stability, and deformation resistance.

### 3.5 Rheological assessment of hybrid oleogels

#### 3.5.1 Temperature ramp test

**Fig. 3:**
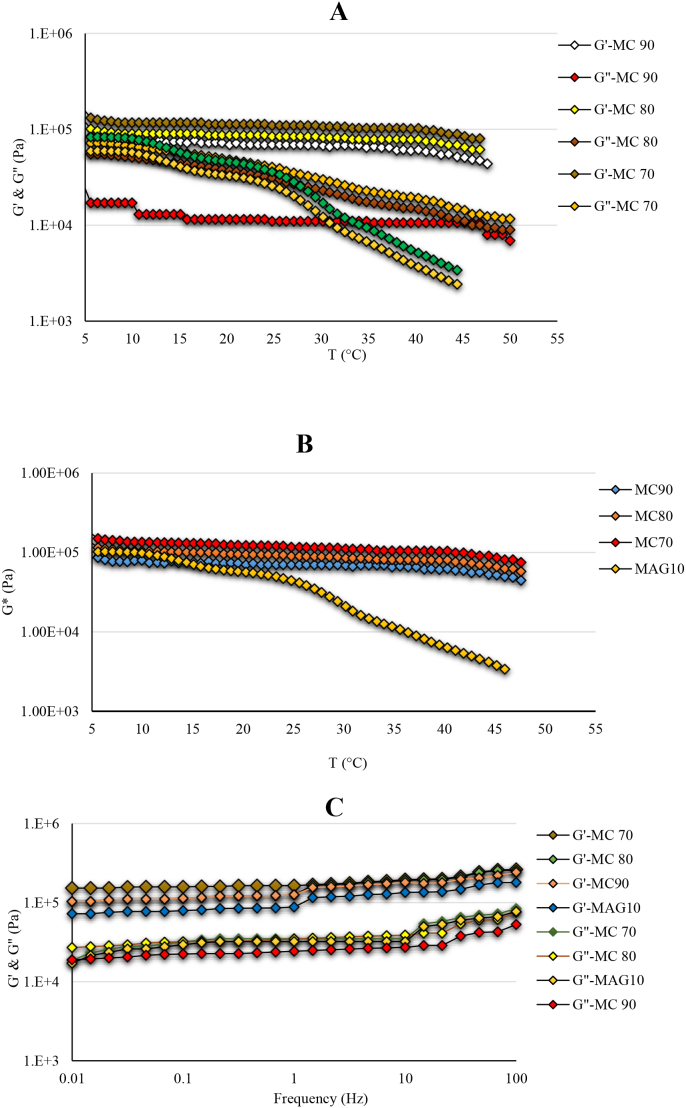
Rheological analysis of hybrid oleogels including sweep temperature test (A), complex modulus (B) and sweep frequency test (C). MC90, MC80, and MC70 are oleogel samples made using oleogelators derived from a W/O emulsion with MAGs: MC ratios of 30:70, 20:80, and 10:90, respectively; MAG10: Control sample. Elastic modulus: (G'), Viscous modulus: (G"), complex modulus: (G*)**.**

The temperature ramp test was employed to evaluate the thermal stability of the hybrid oleogels at 5 to 55 °C. In this test, at constant strain, the fat temperature is raised at a certain rate and their viscous (G') and elastic (G") moduli are evaluated. Fig. 3A shows the curves of G' and G" moduli of the hybrid oleogels. The G' modulus curve of the hybrid oleogels was superior to the G" modulus curve, indicating a prevalent solid-like behavior of the prepared gels (MC70 > MC80 > MC90). Accordingly, the MC70 oleogel had higher G' modulus, compared to control sample, indicating its more solid-like properties (MC70 > MAG10). Fig. 3A also shows that the hybrid oleogels had a good thermostability due to their relatively flat slope at 5 to 55 °C, indicating their acceptable gel strength. Obviously, at higher temperatures, the curves of G" modulus approaches to G', revealing the liquid-like properties. The curve of G' modulus of all hybrid oleogels were nearly vanished at 45 to 50 °C, meaning that the oleogels were slowly melted. As shown in Fig. 3A the curves of G' and G" moduli of control sample (MAG10) showed a different trend compared to hybrid oleogels where they were closer to each other. The curve of G' modulus of MAG10 was disappeared at near 42 °C whereas that of certain hybrid oleogels remained even at 50 °C due to their firmer structure. This result was in accordance with SFC curves where the samples had a low SFC (2–3%) at 50 °C. These findings agreed Patel (2017) who reported that palm stearin/MC-based oleogels were stronger than palm stearin alone at the same concentration.

#### 3.5.2 Complex modulus (G*)

The complex modulus (G*) can be used as a measure of strength or consistency of a structured material. This modulus also indicates the hardness of the samples (Rao, 2010). As shown in Fig. 3B, MC70 had the highest G* modulus among the hybrid oleogels. This result clearly shows that the hybrid oleogels had a greater hardness. They also experienced less changes at higher temperatures (as seen for temperature ramp test). This result was in agreement with the findings of Naderi et al. (2016) who reported that the G* modulus curve of MAG-containing chicken fat possessed a flat slope. Rodríguez-Hernández et al. (2021) reported that MAGs are able to increase the hydrogen bonding of cellulose derivatives in the edible oils. In this context, the main non-covalent interactions include electrostatic interactions, steric exclusion, hydrophobic interactions, and hydrogen bonding. These interactions play a crucial role in the flow, stability, texture, and mouthfeel of oleogels. They also control the interactions between biopolymers and provide textural and sensory properties by creating microstructure in oleogel systems. The importance of these interactions in a specific system depends on factors such as the types of polymers involved, the composition of the liquid mixture, and environmental conditions (Mcclements, 2006).

#### 3.5.3 Frequency sweep tests

This test provides useful information about the viscoelastic properties of the samples as a function of frequency. In this test, G' and G" moduli are examined in a certain frequency range. The fats with higher G' modulus show a lower frequency dependence. In fact, the hybrid oleogels having higher gel strength undergo less changes with frequency variation. As shown in Fig. 3C, the G' modulus curve of the MC70, as reported in the temperature ramp test, had a direct relationship with frequency, but showed a flatter slope (less frequency dependence) than other samples. G' modulus curve of the hybrid oleogels was superior to G" modulus curve, indicating the gel strength of the hybrid oleogels. As illustrated in Fig. 3C, the curves of G' and G" moduli of the control sample had a higher frequency dependence as compared to the hybrid oleogels. In fact, the hybrid oleogels are demonstrated more solid-like nature in comparison with MAG10. The results were consistent with Patel et al., 2014a, Patel et al., 2014b), who found that oleogels made with HPMC and carboxymethyl cellulose (CMC) using the emulsion templated method had a consistent complex modulus curve across temperatures. Their study indicated that lower viscosity cellulose derivatives resulted in oleogels with higher gel strength (G' > 4000 Pa).

The parameters of the power law model (G' = KY^n^) including *K*, *Y* and *n* which show the frequency dependence of the G' modulus of the hybrid oleogels, are listed in Table 1. Correspondingly, the *K* coefficient represents *G'* value at 1 Hz and the n coefficient represents the slope of the relationship between the G' modulus and the frequency. An n value close to zero indicates that the elastic modulus is not frequency dependent, an n value close to 1 indicates that the oleogels have viscous character and a negligible n value shows elastic nature of the hybrid oleogels (Balaghi, Mohammadifar, Zargaraan, Gavlighi, & Mohammadi, 2011). MC70 showed the most *K* coefficient among other samples (*P* < 0.05), demonstrating its elastic character. This result is also confirmed by the negligible *n*-value of the MC70.

**Table 1:** Parameters of power law model including K and n for elastic modulus and 3-ITT Parameters including deformation (%) and recovery (%) of hybrid oleogels.

	Power law model G' = KY^n^			3-ITT Parameters
Treatment	**K (Pa)**	**n**	**R**^**2**^	**Recovery (%)**
MC90	142932^c^	0.10^a^	0.93^a^	37.7^c^
MC80	148790^b^	0.09^a^	0.93^a^	57.2^b^
MC70	178395^a^	0.05^b^	0.82^b^	72.2^a^
MAG10	104153^d^	0.12^a^	0.94^a^	25.1

Different superscripts show significant differences in each column at *p* < 0.05. MC90, MC80, and MC70 are oleogel samples made using oleogelators derived from a W/O emulsion with MAGs:MC ratios of 30:70, 20:80, and 10:90, respectively.; MAG10: Control sample.

#### 3.5.4 Three interval thixotropic test (3-ITT)

Most foods have a thixotropic behavior. Thixotropy is a phenomenon by which the structure of a product is broken down under shear and rebuilt at rest (Steffe, 1996). In other words, this index indicates how some food products can recover their structure after stress (at constant stress or shear rate) (Rao, 2010). Accordingly, this explains why some foods, dilute and flow under stress. In general, two main phenomena are observed in the thixotropic behavior of materials, including structural degradation and structural recovery of the product as a function of time. The 3-interval thixotropy test (3-ITT) examines the structural recovery properties of a product at three different intervals, including two different shear stresses. Typically, the first and the last time intervals are performed at a lower and higher shear rate, respectively.

**Fig. 4:**
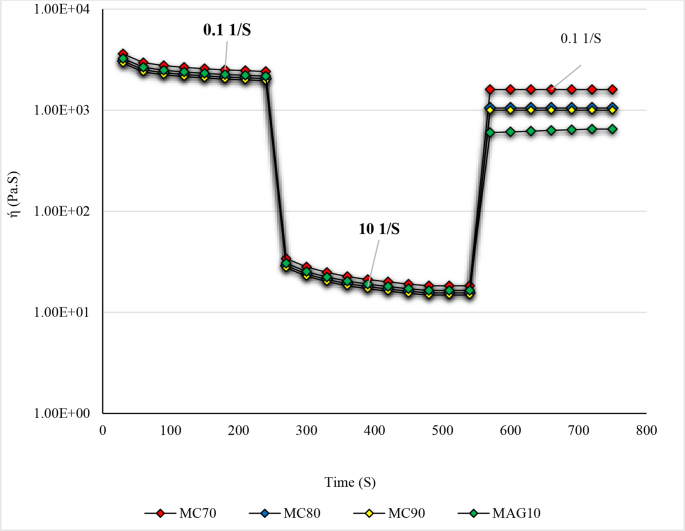
Thixotropic structure recovery curves of hybrid oleogels at low 0.1 s^−1^ and high shear stress 10 s^−1^. MC90, MC80, and MC70 are oleogel samples made using oleogelators derived from a W/O emulsion with MAGs: MC ratios of 30:70, 20:80, and 10:90, respectively; MAG10: Control sample. ή: Viscosity.

Three different phases of the structure recovery curves of the MC90, MC80 and MC70 can be compared in Fig. 4. The MC70 exhibited the highest recovery (%) compared to other samples. Accordingly, higher MAGs in OLGs composition induced higher recovery (%) of hybrid oleogels. The 3-ITT parameter including recovery (%) are given in Table 1. A fat shows a high recovery when the ratio of the maximum viscosity in the third interval to the minimum viscosity in the first interval is around 70% (Ashok R Patel, 2017). As shown in Table 1, the recovery (%) of MC70 was >70%, followed by MC80, MC90 and MAG10. The results indicated that the hybrid oleogels improved the structural properties of MAGs. This could be connected to the reinforcing properties of the polymer strands as suggested by earlier results.

Patel et al., 2014a, Patel et al., 2014b) attributed the acceptable recovery of the hybrid oleogels to the return to the original state of polymer particles that had deformed due to high shear stress in the flow direction. This increases the energy loss and, as a result increase in the resistance to flow. In addition, they found that the degree of viscosity of the cellulose derivatives played a notable role in rheological properties. It was found that MC with a viscosity of 1500 cP was more efficient than that of with a viscosity of 400 cP. The absence of MC caused the least recovery (%) (Table 1). This is probably due to the hydrogen bond formed between MC and MAGs. In fact, MAGs induced an increase in hydrogen bonds in the cellulose derivatives (ethyl cellulose) dissolved in the edible oils and therefore increasing gel strength (Rodríguez-Hernández et al., 2021). This research revealed that with increasing MAGs up to 30% in the hybrid OLGs, the structural strength of the samples increases, while the control with 100% MAGs showed the lowest structural strength. This contradictory observation is due to the lack of MC in control. In fact, cellulose derivatives will be able to increase the structural strength of the crystalline particles by creating a compact system through the formation of hydrogen bonds. Patel et al., 2014a, Patel et al., 2014b) reported recovery rates of 70–90% for MC and HPMC samples, respectively, while Patel and Dewettinck (2015) found weak structure recovery in wax-based hydrogels and HPMC. Differences in results were attributed to the cellulose derivatives used and their viscosity levels leading to the crosslinks formed by MAGs.

## 4 Conclusion

In this study, the potential use of polymers to enhance the gel system formed from crystalline particles was investigated. Monoglycerides (MAGs) have been traditionally used as building blocks for structuring edible oils, resulting in gel systems with limited structural strength. By combining polymer strands with MAGs, it was possible to create gels with increased strength and thermal stability without compromising the oxidative stability of the hybrid oleogels. The hybrid oleogels exhibited a relatively smooth slope in the solid fat content (SFC) curve and showed no significant changes in crystallization kinetics, making them suitable for fat-containing food products requiring high structural integrity.

The results of the temperature ramp test demonstrated the high thermal stability of the hybrid oleogels. Additionally, the frequency sweep test revealed that the hybrid oleogels exhibited less frequency dependence, indicating superior gel strength compared to the control sample. The 3-ITT results showed that the hybrid oleogels had a higher recovery percentage. These findings suggest that the prepared samples could serve as a base stock for the production of shortening and margarine. Overall, the combination of MAGs and polymer strands resulted in enhanced gel properties by accelerating the crystallization process and reinforcing the texture of the final fat product. In this study, MC70 was found to be the most effective treatment. Hybrid oleogels can be utilized to replace commercial shortenings partially. This helps in reducing the SFAs and TFAs content. Shortenings made from lower saturation, with the inclusion of rapeseed oil known for its high unsaturation level, are more preferable.

## CRediT authorship contribution statement

**Mehdi Naderi:** Writing – original draft, Software, Investigation. **Aman Mohammad Ziaiifar:** Writing – review & editing, Supervision, Conceptualization. **Ladan Rashidi:** Writing – review & editing, Visualization, Supervision. **Seid Mahdi Jafari:** Writing – review & editing.

## Declaration of competing interest

The authors declare that they have no known competing financial interests or personal relationships that could have appeared to influence the work reported in this paper.

## Data Availability

The data that has been used is confidential.

## Appendix A Supplementary data

Supplementary material Image 1

## References

[bb0005] Ahmadi P., Tabibiazar M., Roufegarinejad L., Babazadeh A. (2020). Development of behenic acid-ethyl cellulose oleogel stabilized Pickering emulsions as low calorie fat replacer. International Journal of Biological Macromolecules.

[bb0010] Balaghi S., Mohammadifar M.A., Zargaraan A., Gavlighi H.A., Mohammadi M. (2011). Compositional analysis and rheological characterization of gum tragacanth exudates from six species of Iranian Astragalus. Food Hydrocolloids.

[bb0015] Bayés-García L., Patel A.R., Dewettinck K., Rousseau D., Sato K., Ueno S. (2015). Lipid crystallization kinetics—Roles of external factors influencing functionality of end products. Current Opinion in Food Science.

[bb0020] Brühl L. (1997).

[bb0025] Espert M., Salvador A., Sanz T. (2020). Cellulose ether oleogels obtained by emulsion-templated approach without additional thickeners. Food Hydrocolloids.

[bb0030] Garcia C.R., Malik M.H., Biswas S., Tam V.H., Rumbaugh K.P., Li W., Liu X. (2022). Nanoemulsion delivery systems for enhanced efficacy of antimicrobials and essential oils. Biomaterials Science.

[bb0035] Gomes T., Caponio F., Bruno G., Summo C., Paradiso V.M. (2010). Effects of monoacylglycerols on the oxidative stability of olive oil. Journal of the Science of Food and Agriculture.

[bb0040] Grover J.A. (2020). Food hydrocolloids.

[bb0045] Haj Eisa A., Laufer S., Rosen-Kligvasser J., Davidovich-Pinhas M. (2020). Stabilization of ethyl-cellulose Oleogel network using Lauric acid. European Journal of Lipid Science and Technology.

[bb0050] Hwang H.S., Winkler-Moser J.K. (2020). Properties of margarines prepared from soybean oil oleogels with mixtures of candelilla wax and beeswax. Journal of Food Science.

[bb0055] Kim M., Hwang H.-S., Jeong S., Lee S. (2021). Utilization of oleogels with binary oleogelator blends for filling creams low in saturated fat. LWT.

[bb0060] Li L., Liu G. (2023). Engineering effect of oleogels with different structuring mechanisms on the crystallization behavior of cocoa butter. Food Chemistry.

[bb0065] Liu Y., Binks B.P. (2022). Fabrication of stable Oleofoams with Sorbitan Ester surfactants. Langmuir: the ACS journal of surfaces and colloids.

[bb0070] Mcclements D.J. (2006). Non-covalent interactions between proteins and polysaccharides. Biotechnology Advances.

[bb0075] Naderi M., Farmani J., Rashidi L. (2016). Structuring of chicken fat by Monoacylglycerols. Journal of the American Oil Chemists’ Society.

[bb0080] Naderi M., Farmani J., Rashidi L. (2018). The impact of saturated monoacylglycerols on the oxidative stability of Canola oil under various time/temperature conditions. Grasas y Aceites.

[bb0085] Narine S.S., Marangoni A.G. (1999). Relating structure of fat crystal networks to mechanical properties: A review. Food Research International.

[bb0090] O’brien R.D. (2008).

[bb0095] Pan J., Tang L., Dong Q., Li Y., Zhang H. (2021). Effect of oleogelation on physical properties and oxidative stability of camellia oil-based oleogels and oleogel emulsions. Food Research International.

[bb0100] Paradiso V.M., Caponio F., Bruno G., Pasqualone A., Summo C., Gomes T. (2014). Complex role of monoacylglycerols in the oxidation of vegetable oils: Different behaviors of soybean monoacylglycerols in different oils. Journal of Agricultural and Food Chemistry.

[bb0105] Patel A.R. (2017). Methylcellulose-coated microcapsules of palm stearine as structuring templates for creating hybrid oleogels. Materials Chemistry and Physics.

[bb0110] Patel A.R., Cludts N., Bin Sintang M.D., Lewille B., Lesaffer A., Dewettinck K. (2014). Polysaccharide-based oleogels prepared with an emulsion-templated approach. ChemPhysChem.

[bb0115] Patel A.R., Cludts N., Bin Sintang M.D., Lewille B., Lesaffer A., Dewettinck K. (2014). Polysaccharide-based oleogels prepared with an emulsion-templated approach. ChemPhysChem.

[bb0120] Patel A.R., Dewettinck K. (2015). Comparative evaluation of structured oil systems: Shellac oleogel, HPMC oleogel, and HIPE gel. European Journal of Lipid Science and Technology.

[bb0125] Patel A.R., Dewettinck K. (2016). Edible oil structuring: An overview and recent updates. Food & Function.

[bb0130] Qi W., Li T., Zhang Z., Wu T. (2021). Preparation and characterization of oleogel-in-water Pickering emulsions stabilized by cellulose nanocrystals. Food Hydrocolloids.

[bb0135] Rao M.A. (2010).

[bb0140] Rodríguez-Hernández A., Pérez-Martínez J., Gallegos-Infante J., Toro-Vazquez J., Ornelas-Paz J. (2021). Rheological properties of ethyl cellulose-monoglyceride-candelilla wax oleogel Vis-a-Vis edible shortenings. Carbohydrate Polymers.

[bb0145] Saberi A.H., Kee B.B., Oi-Ming L., Miskandar M.S. (2011). Physico-chemical properties of various palm-based diacylglycerol oils in comparison with their corresponding palm-based oils. Food Chemistry.

[bb0150] Sánchez-Becerril M., Marangoni A., Perea-Flores M., Cayetano-Castro N., Martínez-Gutiérrez H., Andraca-Adame J., Pérez-Martínez J. (2018). Characterization of the micro and nanostructure of the candelilla wax organogels crystal networks. Food Structure.

[bb0155] Singh A., Auzanneau F.-I., Rogers M. (2017). Advances in edible oleogel technologies–a decade in review. Food Research International.

[bb0160] Steffe J.F. (1996).

[bb0165] Sun H., Xu J., Lu X., Xu Y., Regenstein J.M., Zhang Y., Wang F. (2022). Development and characterization of monoglyceride oleogels prepared with crude and refined walnut oil. Lwt.

[bb0170] Wang C.S., Virgilio N., Wood-Adams P., Heuzey M.C. (2017). A mechanism for the synergistic gelation properties of gelatin B and xanthan gum aqueous mixtures. Carbohydrate Polymers.

[bb0175] Wei F., Lu M., Li J., Xiao J., Rogers M.A., Cao Y., Lan Y. (2022). Construction of foam-templated oleogels based on rice bran protein. Food Hydrocolloids.

[bb0180] Yousefi S., Rajaei P., Nateghi L., Rashidi Nodeh H., Rashidi L. (2023). Encapsulation of sesamol and vitamin A using alginate and chitosan-coated W/O/W multiple emulsions containing Tween 80 and Span 80. International Journal of Biological Macromolecules.

[bb0185] Zhang Y., Xu J., Tang C., Li Y. (2023). Crystallization behavior and physical properties of monoglycerides-based oleogels as function of oleogelator concentration. Foods.

