# Supplementary material for: Rapeseed oleogels based on monoacylglycerols and methylcellulose hybrid oleogelators: Physicochemical and rheological properties
Source: Food Chem X. 2024 May 28;23:101520. doi: 10.1016/j.fochx.2024.101520 (PMC11200285; doi:10.1016/j.fochx.2024.101520)
Supplement: Supplementary file 1 — Supplementary material [file mmc1.docx]

**Table 1S.** Rancimat Test (**(110 °C)**) and Liquid phase (%) of methylcellulose-based hybrid oleogel at 5, 25 and 45**°**C.

| **Treatment** | **Rancimat test (110 °C)** | **Liquid phase (%)** | | |
| --- | --- | --- | --- | --- |
|  |  | **5°C** | **25°C** | **45°C** |
| **MC90** | 28.2±0.8^b^ | 28.2±0.8^b^ | 47.4±0.8^b^ | 58.1±0.8^b^ |
| **MC80** | 27.9±0.9^b^ | 27.9±0.9^b^ | 45.4±0.7^c^ | 54.8±0.9^c^ |
| **MC70** | 21.2±0.8^c^ | 21.2±0.8^c8^ | 28.5±0.8^d^ | 38.4±0.7^d^ |
| **MAG10** | 28.7±0.7^a^ | 28.7±0.7^a^ | 48.2±0.7^a^ | 63.2±0.7^a^ |

Numbers are shown as the average of 3 replicates SD. Different superscripts show significant differences in each column at p<0.05. MC90, MC80, and MC70 are oleogel samples made using oleogelators derived from a W/O emulsion with MAGs:MC ratios of 30:70, 20:80, and 10:90, respectively. ; MAG10: Control sample.

**Table 2S.** The SFC (%) of MC-based hybrid oleogels at 5 to 45 °C

| Treatment | SFC (%) | | | | |
| --- | --- | --- | --- | --- | --- |
|  | **5 °C** | **10 °C** | **25 °C** | **35 °C** | **45 °C** |
| MC90 | 9.6±0.1^Aa^ | 9.3±0.1^Aa^ | 7.6±0.1^Ab^ | 6.3±0.1^Ab^ | 4.5±0.1^Ac^ |
| MC80 | 9.8±0.1^Aa^ | 9.6±0.1^Aa^ | 7.7±0.1^Ab^ | 6.5±0.1^Ab^ | 4.8±0.1^Ac^ |
| MC70 | 9.9±0.1^Aa^ | 9.8±0.1^Aa^ | 7.8±0.1^Ab^ | 6.7±0.1^Ab^ | 4.9±0.1^Ac^ |
| MAG10 | 10.1±0.1^Aa^ | 9.9±0.1^Aa^ | 8.1±0.1^Ab^ | 6.8±0.1^Ab^ | 5.0±0.1^Ac^ |

Numbers are shown as the average of 3 replicates SD. Numbers with the same uppercase and lowercase letters in each column and row indicated no significance at P<0.05. MC90, MC80, and MC70 are oleogel samples made using oleogelators derived from a W/O emulsion with MAGs:MC ratios of 30:70, 20:80, and 10:90, respectively. ; MAG10: Control sample.

**Table 3S.** Induction priod of crystallization (IP_CRYST_) and crystallization rates of hybrid oleogels at 0°C, 5 °C, 25 °C and 45 °C

| T (°C) | Treatment | Crystallization kinetics | |
| --- | --- | --- | --- |
|  |  | IP_Cryst_ _(S )_ | crystallization Rate _(1/min)_ |
| 0 °C | MC90 | 0.50±0.06 ^a^ | 0.67±0.06 ^a^ |
|  | MC80 | 0.30±0.06 ^ab^ | 0.82±0.06 ^ac^ |
|  | MC70 | 0.10±0.06 ^b^ | 0.91±0.06 ^b^ |
|  | MAG10 | 0.02±0.06 ^c^ | 0.98±0.06 ^c^ |
|  |  |  |  |
| 5 °C | MC90 | 0.70±0.06 ^a^ | 0.53±0.06 ^a^ |
|  | MC80 | 0.60±0.06 ^b^ | 0.68±0.06 ^b^ |
|  | MC70 | 0.50±0.06 ^bc^ | 0.77±0.06 ^bc^ |
|  | MAG10 | 0.42±0.06 ^c^ | 0.84±0.06 ^c^ |
|  |  |  |  |
| 25 °C | MC90 | 1.90±0.06 ^a^ | 0.49±0.06 ^a^ |
|  | MC80 | 1.40±0.06 ^ab^ | 0.64±0.06 ^b^ |
|  | MC70 | 1.10±0.06^bc^ | 0.73±0.06 ^bc^ |
|  | MAG10 | 1.02±0.06 ^c^ | 0.8±0.06 ^c^ |
|  |  |  |  |
| 45 °C | MC90 | 11.70±0.06 ^a^ | 0.24±0.06 ^a^ |
|  | MC80 | 10.50±0.06 ^b^ | 0.39±0.06 ^b^ |
|  | MC70 | 9.80±0.06 ^c^ | 0.48±0.06 ^bc^ |
|  | MAG10 | 9.72±0.06 ^c^ | 0.55±0.06 ^c^ |

Numbers are shown as the average of 3 replicates SD. Different superscripts showed significant differences in each temperature/column at p<0.05. MC90, MC80, and MC70 are oleogel samples made using oleogelators derived from a W/O emulsion with MAGs:MC ratios of 30:70, 20:80, and 10:90, respectively. ; MAG10: Control sample.


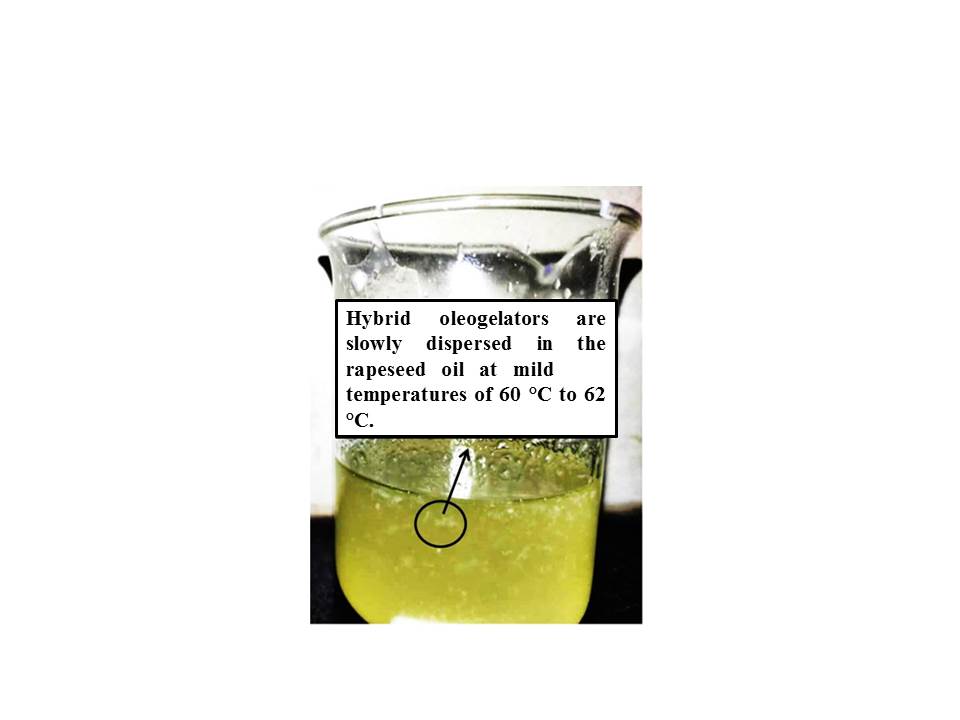


**B**

**A**

**Figure 1S.** An emulsion structured with MAG (30% (w/w)) and an aqueous solution of 2% (w/w) MC (70%) (A); MC70 Oleogel (B).
